# Supplementary material for: Identification of photoperiod-induced specific miRNAs in the adrenal glands of Sunite sheep (Ovis aries)
Source: Front Vet Sci. 2022 Jul 22;9:888207. doi: 10.3389/fvets.2022.888207 (PMC9354845; doi:10.3389/fvets.2022.888207)
Supplement: Supplementary file 1 [file Data_Sheet_1.ZIP › Supplementary file/miRDeep2 file.zip!/miRDeep2 file/Know_Structure/LP42A1/oar-mir-148a.pdf]

miRBase precursor : oar-mir-148a
Total read count : 1810042
oar-miR-148a read count : 1806939
remaining reads : 3103

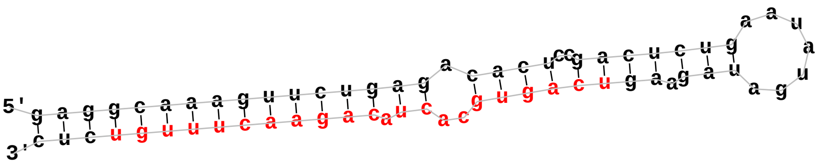

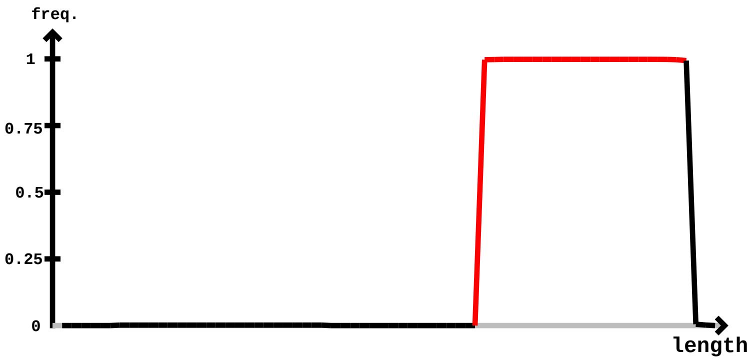

oar-miR-148a

```
5'- gaggcaaaguucugagacacuccgacucugaauaugauagaagucagugcacuacagaacuuugucuc  -3'   exp
    ((((((((((((((((.((((..(((((((.......))).))))))))..)).))))))))))))))  reads     mm      sample
    ....caaaguucugagacacuccga...........................................  4         0       seq
    ....caaaguucugagacacuccgac..........................................  3         0       seq
    ....caaaguucugagacacuccgacu.........................................  74        0       seq
    .....aaaguucugagacacuccg............................................  6         0       seq
    .....aaaguucugagacacuccga...........................................  72        0       seq
    .....aaaguucugagacacuccgac..........................................  31        0       seq
    .....aaaguucugagacacuccgacu.........................................  2897      0       seq
    .....aaaguucugagacacuccgacuc........................................  7         0       seq
    .....aaaguucugagacacuccgacucu.......................................  6         0       seq
    ......aaguucugagacacuccgacu.........................................  3         0       seq
    ..........................................gucagugcacuacagaacuuug....  1         0       seq
    ..........................................gucagugcacuacagaacuuugu...  80        0       seq
    ..........................................guUagugcacuacagaacuuugu...  1         1       seq
    ...........................................ucagugcacuacagaac........  8         0       seq
    ...........................................ucaCugcacuacagaacu.......  3         1       seq
    ...........................................uUagugcacuacagaacu.......  1         1       seq
    ...........................................ucagugcacuacagaacu.......  156       0       seq
    ...........................................ucagugcacuacagaacuu......  283       0       seq
    ...........................................ucaguAcacuacagaacuu......  1         1       seq
    ...........................................uUagugcacuacagaacuuu.....  3         1       seq
    ...........................................ucagugcacuacagaacuuu.....  2006      0       seq
    ...........................................uUagugcacuacagaacuuug....  5         1       seq
    ...........................................ucagugcacuacagaacuuug....  6015      0       seq
    ...........................................uUagugcacuacagaacuuugu...  2603      1       seq
    ...........................................ucagugcacuacagaacuuugu...  1784016   0       seq
    ...........................................ucagugcacuacagaacuuuguc..  5719      0       seq
    ...........................................uUagugcacuacagaacuuuguc..  8         1       seq
    ...........................................ucagugcacuacagaacuuugucu.  3535      0       seq
    ...........................................ucagugcacuacagaacuuugucuc  17        0       seq
    ............................................cagugcacuacagaacuuu.....  1         0       seq
    ............................................cagugcacuacagaacuuug....  1         0       seq
    ............................................cagugcacuacagaacuuugu...  989       0       seq
    ............................................Uagugcacuacagaacuuugu...  4         1       seq
    ............................................cagugcacuacagaacuuuguc..  11        0       seq
    ............................................cagugcacuacagaacuuugucu.  5         0       seq
```

oar-miR-148a

```
gaggcaaaguucugagacacuccgacucugaauaugauagaagucagugcacuacagaacuuugucuc
............................................agugcacuacagaacuuug....    3       0       seq
............................................agugcacuacagaacuuugu...    1444    0       seq
............................................agugcacuacagaacuuuguc..    5       0       seq
............................................agugcacuacagaacuuugucu.    2       0       seq
............................................agugcacuacagaacuuugucC.    2       1       seq
.............................................gugcacuacagaacuuugu...    2       0       seq
..............................................ugcacuacagaacuuugu...    1       0       seq
...............................................gcacuacagaacuuugu...    8       0       seq
```
